# Successful Management of Status Asthmaticus via Veno‐Venous ECMO: Extubation Prior to Decannulation—A Case Report

**DOI:** 10.1155/crcc/7781487

**Published:** 2026-04-29

**Authors:** Sheryl S. Ang

**Affiliations:** ^1^ Department of Anesthesiology and Perioperative Medicine, Medical College of Georgia, Augusta University, Augusta, Georgia, augusta.edu

**Keywords:** management, status asthmaticus, veno-venous ECMO

## Abstract

**Background:**

The use of veno‐venous extracorporeal membrane oxygenation (VV‐ECMO) has risen exponentially since the COVID pandemic. VV‐ECMO has been utilized successfully to manage patients in status asthmaticus who have failed to respond adequately to conventional therapeutic interventions and mechanical ventilatory support. However, clear guidelines on the management of mechanical ventilation for these patients while on VV‐ECMO support appear to be lacking.

**Case Presentation:**

A 52‐year‐old male was placed on emergent VV‐ECMO for status asthmaticus after developing worsening hypercarbic respiratory failure despite mechanical ventilation and aggressive bronchodilator therapy. Once he was stabilized on VV‐ECMO, there was a dilemma in management priorities—extubate the patient while keeping full VV‐ECMO support versus working to aggressively wean VV‐ECMO support while the patient is intubated. Ultimately, the decision was made to extubate the patient about 60 h postcannulation. Postexubation, however, he was in respiratory distress with stridor and worsening bronchospasm and required maximum sweep on the VV‐ECMO in addition to increased respiratory support via noninvasive ventilation (NIV). Ultimately, he turned the corner, made progressive improvement, was weaned from VV‐ECMO support, and was decannulated 7 days after cannulation.

**Conclusion:**

Extubating a patient placed on VV‐ECMO for status asthmaticus is not a straightforward decision. One needs to consider the management priorities, anticipate the potential problems, and understand the implications of early extubation.

## 1. Introduction

Asthma is a prevalent disorder among children and adults, with approximately 7% of the United States population reporting a current diagnosis of asthma [1]. Status asthmaticus accounts for approximately 1.9% of all causes of acute respiratory failure requiring veno‐venous extracorporeal membrane oxygenation (VV‐ECMO) support [2]. VV‐ECMO has been utilized successfully to manage patients in status asthmaticus who have failed to respond adequately to conventional therapeutic interventions and mechanical ventilatory support. It is found that status asthmaticus may be associated with greater survival than other indications for extracorporeal life support (ELS) [2]. The average time on VV‐ECMO for patients with status asthmaticus can range anywhere between 36 and 384 h [3]. There currently exist no clear guidelines as to the timing of extubation in these patients postcannulation. In a recently published case report, the approach used was to liberate the patient from VV‐ECMO prior to extubation [4]. Being on the ventilator allows day‐to‐day monitoring of respiratory parameters such as the peak airway pressure and pressure‐volume loop, which indicates the severity of a patient′s bronchospasm and provides the care team with valuable information to anticipate the trajectory of a patient′s status asthmaticus. However, early extubation will promote early mobility, which has been shown to shorten length of stay in the intensive care unit and improve functional mobility [5]. Thus, the timing of extubation of the patient while on VV‐ECMO is a complex one. In this case, we describe a patient extubated 60 h after emergent VV‐ECMO cannulation for status asthmaticus who struggled from a respiratory standpoint in the initial postextubation phase. It created a management dilemma: whether to wean VV‐ECMO support or to liberate him from the ventilator first.

## 2. Case Description

We describe a 52‐year‐old morbidly obese African American male who presented to the emergency department (ED) with shortness of breath, which has been progressively worsening over the past few weeks, associated with upper respiratory illness symptoms. His comorbidities include asthma, hypertension, heart failure with mildly reduced ejective fraction (40%–45%), and type II diabetes mellitus. Of note, he had run out of his asthma medications recently. His vital signs on presentation were as follows: blood pressure (BP) 179/110 mmHg, heart rate (HR) 106 beats per minute (bpm), respiratory rate (RR) 22 per minute, and pulse oximetry (SpO2) 90% on room air. He had an audible bilateral expiratory wheeze and increased work of breathing. He was diagnosed with a moderate asthma exacerbation based on the above clinical signs. SpO2 improved to 94% on oxygen supplementation via nasal cannula at 3 liters per minute (L/min). He was given albuterol and ipratropium bromide nebulization treatment twice and a dose of intravenous dexamethasone 10 mg. Posttreatment, over the 16‐h observation period in the ED, he showed clinical improvement with reduced shortness of breath and diminished wheezing, and he was weaned off oxygen. Hence, he was discharged with albuterol refills, a 2‐day follow‐up with family medicine and a referral to outpatient pulmonology. On discharge, his vital signs were BP 114/88 mmHg, HR 93 bpm, RR 22 per minute, and SpO2 was 92% on room air.

He re‐presented to the ED 2 days later with worsening symptoms and hypoxia to 85% on room air. He was diagnosed with worsening asthma exacerbation and started on methylprednisone, albuterol, and ipratropium bromide nebulization, and noninvasive ventilation (NIV), then admitted to the medical intensive care unit. A ketamine drip was started to optimize bronchodilation. He tested positive for influenza A. Despite increasing support on NIV with intermittent high flow nasal cannula, his hypercarbia continued to worsen with increasing work of breathing. He was intubated on the third day after presentation when he became obtunded due to severe hypercarbia. At that time, arterial blood gas showed a pH of 7.23, pCO_2_ 83.5 mmHg, pO_2_ 126 mmHg, HCO_3_ 35.2 mmol/L on an FiO_2_ of 50%. While on the ventilator, there were persistently high peak airway pressures of up to 50 cmH_2_O and worsening respiratory acidosis despite continuous albuterol treatment. Heliox was started, and the patient was sedated and paralyzed. Despite these interventions, his respiratory acidosis continued to worsen. The partial pressure of carbon dioxide (pCO2) on arterial blood gas was up to 86 mmHg with a pH of 7.19. He required hemodynamic support with norepinephrine at 0.12 mcg/kg/min. Cardiothoracic surgery was consulted for consideration of VV‐ECMO for hypercarbic respiratory failure despite maximal ventilatory support, and he was deemed a good candidate.

**Figure 1:**
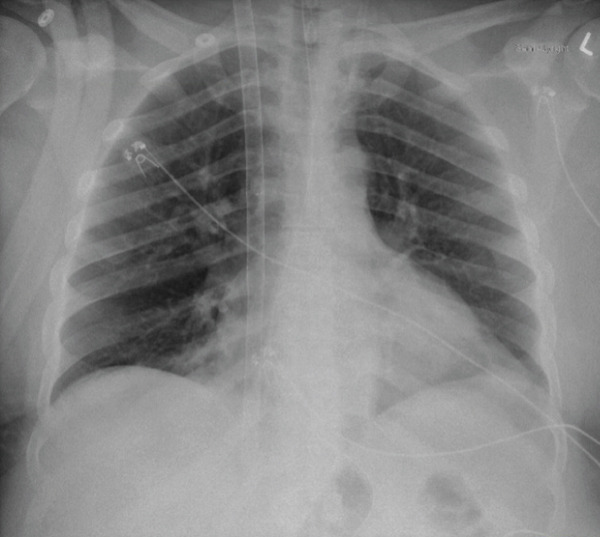
Chest x‐ray postcannulation showing the presence of the right internal jugular vein VV‐ECMO cannula, with largely clear lung fields bilaterally.

**Figure 2:**
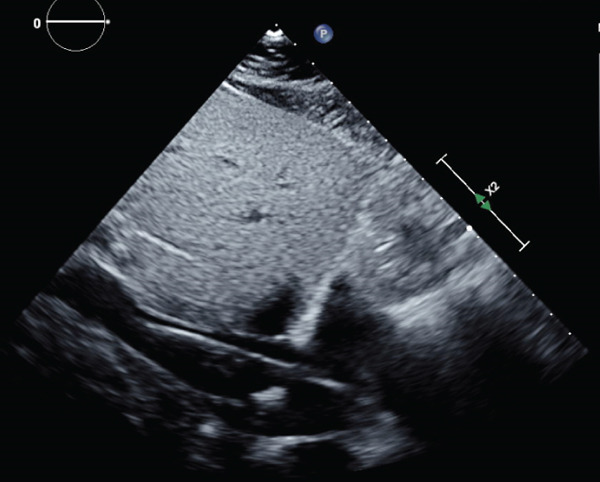
Subcostal view on transthoracic echocardiogram showing distal VV‐ECMO cannula in the inferior vena cava.

He was brought to the operating room emergently for VV‐ECMO cannulation within 24 h after intubation. A right internal jugular 30Fr Crescent Medtronic cannula was placed under transesophageal echocardiogram (TEE) guidance (see Figures 1 and 2). The midesophageal bicaval view was used to ensure optimal cannula positioning with the return port in the right atrium and directed toward the tricuspid valve. Initial VV‐ECMO settings were as follows: fraction of delivered oxygen of 100%, speed of 3600 revolutions per min (RPM), flow of 4.2 L/min, and sweep of 6 L/min. Intraoperative TEE showed a left ventricular ejection fraction of 50% and mildly reduced right ventricular function with no significant valvular pathology. He was transported to the cardiovascular intensive care unit (CVICU) postoperatively. Postcannulation arterial blood gases showed significant improvement in his hypercarbia; pCO2 was down to 52 mmHg with a pH of 7.35. He was initially sedated with dexmedetomidine and hydromorphone and was continued on aggressive bronchodilator therapy including methylprednisolone, continuous albuterol nebulization, scheduled inhaled ipratropium bromide, ketamine infusion, and magnesium. He had persistent sinus tachycardic up to a HR of 130 bpm at first, which improved with downtitration of the dose of ketamine and continuous albuterol nebulization. He was subsequently weaned off sedation on the first day postcannulation, and mental status was ascertained to be intact. Systemic anticoagulation was initiated using intravenous heparin.

**Figure 3:**
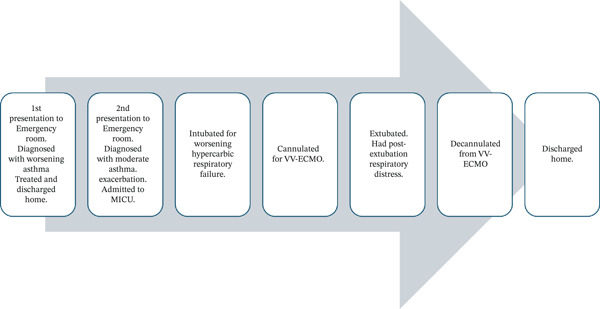
Sequence of events.

The patient continued to show adequate oxygenation and ventilation on stable VV‐ECMO settings on the second day postcannulation and was extubated approximately 60 h after cannulation, with the aim of early mobilization on VV‐ECMO while awaiting lung recovery. At the time of extubation, his RR was 20–30 per minute with tidal volumes around 250 mL on pressure control mode of ventilation with an FiO2 of 40%. A rapid shallow breathing index was not measured as the decision for extubation was irrespective of whether he met extubation criteria or not. The rationale was that he was fully dependent on the VV‐ECMO for oxygenation and ventilation support. However, immediately after extubation, he developed respiratory distress with stridor and worsening tachypnea. NIV was administered preemptively to assist with his work of breathing. He was also given a dose of racemic epinephrine due to the concern for airway edema. The sweep on the VV‐ECMO circuit was increased from 5 to a maximum of 10 L/min. While on NIV, he had an episode of emesis and was transitioned to high flow nasal cannula to minimize the risk of aspiration. At one point, there was a concern that the patient may need to be reintubated for respiratory distress. The maintenance of adequate oxygenation and ventilation in this patient was made more challenging as the patient was morbidly obese, and the VV‐ECMO flow may be insufficient relative to the patient′s cardiac output and body surface area. However, increasing the speed to achieve a higher flow rate was not an option, since the venous pressure of the circuit was close to the safe limit of negative 100 mmHg. Initial blood gas postextubation showed an increase in pCO2 from the 30s to the 50s, but pH remained in the normal range. Fortunately, the patient′s work of breathing improved over the course of the day while continued on the abovementioned bronchodilator therapy, and the sweep on the VV‐ECMO was able to be gradually weaned back down to 6 L/min. Over the next 4 days, with continued aggressive bronchodilator therapy, the VV‐ECMO sweep was weaned down to 1, and he was able to be mobilized out of bed to chair, participate in physical therapy, and took a few steps around his room. He tolerated a capping trial on the sixth day postcannulation and was decannulated on the seventh day uneventfully (see Figure 3).

## 3. Discussion

This case highlights the dilemma regarding priorities in the management of a patient cannulated for VV‐ECMO secondary to status asthmaticus. On one hand, it is ideal for a patient to be extubated while on VV‐ECMO, while waiting for the lungs to recover, as early mobility has definite benefits. On the other hand, weaning off the VV‐ECMO may arguably take precedence since ELS is a risk factor for complications such as thrombosis, bleeding, hemolysis, and vascular injuries [6]. It is well‐documented in the literature that awake, nonintubated patients on VV‐ECMO for chronic respiratory failure as a bridge to lung transplant benefit from the reduction in ventilator‐associated complications and early mobilization [7]. There is also evidence from a retrospective analysis that patients with acute respiratory distress syndrome who are liberated from positive pressure ventilation while on ELS have a significantly higher number of ventilator‐free days and improved rate of survival to hospital discharge, compared with those who were not [8]. However, there is little, if any, evidence in the literature about the ideal management strategy of a patient put on VV‐ECMO specifically for status asthmaticus. This may be contributed by the difficulty in predicting the trajectory of someone in status asthmaticus. Evidence has shown that the mean time spent on VV‐ECMO for a status asthmaticus patient is approximately 12.5 days but could be as little as 36 h or as long as 16 days [3]. In other words, a patient can turn around quickly or take days or weeks to recover. In a patient whose bronchospasm breaks quickly, one approach would be to focus on weaning the VV‐ECMO with the aim of decannulating the patient as early as possible, while providing ventilator support. Conversely, if the patient is slow to recover, then perhaps the approach of early liberation from the ventilator, accompanied by early mobilization may yield more benefit. It is a challenging decision since it is difficult to predict who will fall into each category. When deciding to extubate a patient while on VV‐ECMO, there are additional considerations such as possible increased work of breathing postextubation, as demonstrated by the case described, or inadequate oxygenation and ventilation. In such a situation, additional respiratory support on top of being on full VV‐ECMO support may be necessary, especially in the obese patient. This case demonstrates successful management of a patient in status asthmaticus requiring VV‐ECMO with the approach of early extubation and mobilization before decannulation. Further research is required to determine which patients would benefit from this approach.

## 4. Conclusion

The timing of extubation of patients cannulated for VV‐ECMO due to status asthmaticus is a challenging decision. Key considerations include the anticipated disease trajectory and the careful weighing of the risks versus benefits of early liberation from the ventilator versus early liberation from VV‐ECMO support. Ideally, the decision should be tailored to the individual patient and involve a multidisciplinary discussion to reach a consensus.

## Funding

No funding was received for this manuscript.

## Consent

No written consent has been obtained from the patient, as there is no patient‐identifiable data included in this case report.

## Conflicts of Interest

The author declares no conflicts of interest.

## Data Availability

Data sharing is not applicable to this article as no datasets were generated or analyzed during the current study.
